# The fallacy of enrolling only high-risk subjects in cancer prevention trials: Is there a "free lunch"?

**DOI:** 10.1186/1471-2288-4-24

**Published:** 2004-10-04

**Authors:** Stuart G Baker, Barnett S Kramer, Donald Corle

**Affiliations:** 1 Biometry Research Group, Division of Cancer Prevention, National Cancer Institute, Bethesda, Maryland, USA; 2 Office of Disease Prevention, National Institutes of Health, Bethesda, Maryland, USA

## Abstract

**Background:**

There is a common belief that most cancer prevention trials should be restricted to high-risk subjects in order to increase statistical power. This strategy is appropriate if the ultimate target population is subjects at the same high-risk. However if the target population is the general population, three assumptions may underlie the decision to enroll high-risk subject instead of average-risk subjects from the general population: higher statistical power for the same sample size, lower costs for the same power and type I error, and a correct ratio of benefits to harms. We critically investigate the plausibility of these assumptions.

**Methods:**

We considered each assumption in the context of a simple example. We investigated statistical power for fixed sample size when the investigators assume that relative risk is invariant over risk group, but when, in reality, risk difference is invariant over risk groups. We investigated possible costs when a trial of high-risk subjects has the same power and type I error as a larger trial of average-risk subjects from the general population. We investigated the ratios of benefit to harms when extrapolating from high-risk to average-risk subjects.

**Results:**

Appearances here are misleading. First, the increase in statistical power with a trial of high-risk subjects rather than the same number of average-risk subjects from the general population assumes that the relative risk is the same for high-risk and average-risk subjects. However, if the absolute risk difference rather than the relative risk were the same, the power can be less with the high-risk subjects. In the analysis of data from a cancer prevention trial, we found that invariance of absolute risk difference over risk groups was nearly as plausible as invariance of relative risk over risk groups. Therefore *a priori *assumptions of constant relative risk across risk groups are not robust, limiting extrapolation of estimates of benefit to the general population. Second, a trial of high-risk subjects may cost more than a larger trial of average risk subjects with the same power and type I error because of additional recruitment and diagnostic testing to identify high-risk subjects. Third, the ratio of benefits to harms may be more favorable in high-risk persons than in average-risk persons in the general population, which means that extrapolating this ratio to the general population would be misleading. Thus there is no free lunch when using a trial of high-risk subjects to extrapolate results to the general population.

**Conclusion:**

Unless the intervention is targeted to only high-risk subjects, cancer prevention trials should be implemented in the general population.

## Background

Some prevention trials are restricted to high-risk subjects. If the investigators are only interested in the effects of the intervention on subjects at increased risk [1] or if the study is designed to be a preliminary investigation in preparation for a definitive study in the general population, we think this restriction is reasonable.

However some investigators who are interested in studying the effect of the intervention in the general population may be tempted to design a "definitive" study to estimate the effect of the intervention in a high-risk group. Some investigators may believe that a trial of high-risk subjects would have greater power than a trial of the same size among average-risk subjects. Some examples of this type of thinking can be found in papers on risk prediction models [2,3]. Some investigators may believe that a trial of high-risk subjects with the same power as a trial of average-risk subjects would have lower costs than a trial of average-risk subjects. Some investigators may believe the ratio of benefits to harms can be correctly extrapolated from high-risk to average-risk subjects. Although the rationales for these various beliefs are related, they involve some distinct underlying assumptions that are important to critically examine.

## Methods and results

### Possibly lower statistical power

To crystallize our thinking about statistical power, we consider the following simple hypothetical and realistic example. Investigators want to estimate the effect of intervention in the general population, so they first consider designing a randomized trial among the general at-risk population. Suppose they anticipate that the cumulative probability of incident cancer over the course of the study is *p*_*C *_= .02 in the control arm and *p*_*I *_= .01 in the study arm, and they believe that the difference in probabilities is clinically significant. Also suppose that due to the limited availability of the intervention, they can enroll at most *n *= 2000 study participants in each arm. The investigators compute power using the following standard formula [1] setting the two-sided type I error at .05,



where NormalCDF is the cumulative distribution function for a normal distribution with mean 0 and variance 1, Δ is the anticipated difference one wants to detect, *n *is the sample size per arm, *se*_*Null *_is the standard error under the null hypothesis, and *se*_*Alt *_is the standard error under the alternative hypothesis. Let *p *= (*p*_*C *_+ *p*_*I*_)/2. As discussed in [1], for a study designed to estimate the absolute risk difference, the statistic of interest is , so



For a study designed to estimate the relative risk, the statistic of interest is , so



Applying these formulas to the above example and substituting either (2) or (3) into (1), the investigators obtain a power of .74 based on the absolute risk difference statistic and a power .76 based on a relative risk statistic [see Additional file 1].

Suppose the investigators think this power is too low. To increase power they propose to restrict the study to a high-risk group in which the probability of cancer is .04. Also suppose the investigators make the typical assumption that if the intervention yields a relative risk of .5 in the general population, it would also yield a relative risk of .5 in the high-risk group. Applying (1–3) with high risk subjects for whom *p*_*C *_= .04 and *p*_*I *_= .02 with *n *= 2000, the investigators compute a power of .96 using either the absolute risk difference or relative risk. Because the power is higher using high-risk subjects, the investigators plan the study for a high-risk population and will generalize the results to the general population.

Is there a free lunch? An underlying assumption in this example is that the relative risk is invariant between the general population and the high-risk group. There is no free lunch because the impact of violating this assumption could be substantial. For example, suppose instead that the absolute risk difference is invariant between the general population and the high risk group. Under this scenario the absolute risk difference in the general population is .01, so the absolute risk difference in the high-risk group is also .01. In this case for *p*_*C *_= .04, *p*_*I *_= .03, and *n *= 2000, the power (computed using either absolute risk difference or relative risk statistics) for the trial of high-risk subjects is only .41. The decreased power in a high risk group under a constant risk difference model is not surprising: if the risk difference *p*_*C *_- *p*_*I *_is the same, but *p*_*I *_is increasing, the variances, *p*_*C*_(1 - *p*_*C*_)/*n *and *p*_*I*_(1 - *p*_*I*_)/*n*, will increase as *p*_*C *_increases up to .5, which will reduce the power.

A crucial issue is whether or not the absolute risk difference or the relative risk is likely invariant between average-risk subjects in the general population and high-risk subjects. The answer depends on the cancer, the interventions, and the biology. To gain some appreciation of this issue, we analyzed published data (summarized in Table 1) from a prevention trial of particular interest to us, a study of tamoxifen for the prevention of breast cancer [5]. Rather than limit the analysis to one particular high-risk group, we investigated subjects at various levels of risk defined separately by three variables: age, predicted risk, (the five-year risk of cancer based on the Gail model [3]), and family risk. We fit four models separately to each variable:

**Table 1:** Data from a cancer prevention trial for investigating assumptions of constant risk difference and relative risk when risk groups change.

			Placebo group		Tamoxifen group	
Variable	risk group		cancer	at risk	cancer	at risk
age at entry	1	≤ 49	68	10149	38	10045
	2	50–59	50	7912	25	8040
	3	>60	57	7719	26	7782
predicted risk	1	≤ 2.00%	35	6318	13	6311
	2	2.01–3.01%	42	8108	29	8262
	3	3.01–5.00%	43	7313	27	6959
	4	≤ 5.01%	55	4142	20	4425
family risk	1	0	38	5891	17	5724
	2	1	90	15000	46	15182
	3	2	37	4263	20	4211
	4	3	10	729	6	855

Cancer is invasive breast cancer. Predicted risk is the 5-year predicted risk. Family risk is number of first degree relatives with breast cancer. Data are from Table 5 of [5] with number at risk computed by dividing number of breast cancers by reported breast cancer rate.

constant risk difference,



where *δ *is the risk difference that is constant over groups;

varying risk difference,



where *δ*_*i *_is the risk difference that varies over groups;

constant relative risk,



where *β *is the relative risk that is constant over groups;

varying relative risk,



where *β *is the relative risk that varies over groups.

We obtained maximum likelihood estimates of *δ*, *δ*_*i*_, *β*, and *β*_*i *_using a Newton-Raphson procedure [see Additional file 2].

**Figure 1:**
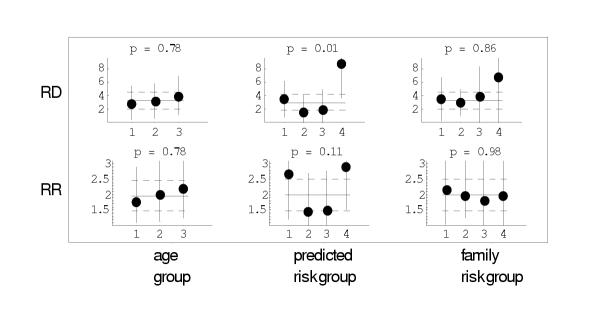
Data from the tamoxifen prevention trial. See text for a description of groups. Horizontal lines are estimates and 95% confidence intervals for model for constant absolute risk difference per 1000 (RD) or relative risk (RR). P-values correspond to likelihood ratio tests comparing the models with varying and constant risk difference or relative risks.

To investigate the plausibility of the constant relative risk and constant risk difference models in this example, we plotted the estimates of *δ*, *δ*_*i*_, *β*, and *β*_*i *_along with confidence intervals (Figure 1). In the top row of Figure 1 we plotted points corresponding to  with (100 - 5/*k*) % confidence intervals and horizontal lines for  with 95% confidence intervals. We also presented the p-values corresponding to twice the difference in log-likelihoods for *Varying RD *versus *Constant RD*. Similarly, in the bottom row of Figure 1, we plotted points corresponding to  with (100 - 5/*k*)% confidence intervals and horizontal lines for  with 95% confidence intervals. We also presented the p-value corresponding to twice the difference in log-likelihoods for *Varying RR *versus *Constant RR*. Out of 6 p-values (3 risk factors × 2 statistics) only one, for absolute risk difference under the risk factor of predicted risk had a small p-value (and the p-value of .01 would not be significant at the .05 level under a Bonferroni adjustment of .05/6). Based on these p-values and inspection of Figure 1, the models *Constant RD *and *Constant RR *are both plausible, especially for age and family risk.

The trial designer does not know the true state of nature. If *Constant RD *is the true state of nature, the power will be *lower *in the high-risk group than the general population. However if *Constant RR *is the true state of nature, the power will be *greater *in the high-risk group than the general population. Thus there is high probability that the power could be reduced when studying high-risk subjects than when studying the general population. Therefore, there is no free lunch in terms of lowering statistical power.

### Possibly increased costs

Even if the model is correct (namely *p*_*C *_and *p*_*I *_are correctly chosen), the smaller trial of high-risk subjects may be more expensive than the larger trial of average-risk subjects from the general population. Consider the following two trials with a power of .90 and a one-sided type I error of .05. In the trial of high-risk subjects *p*_*C *_= .04 and *p*_*I *_= .02, and in the trial of average-risk subjects, *p*_*C *_= .02 and *p*_*I *_= .01. Suppose the statistic of interest is the absolute risk difference. To obtain sample size for each randomization group we use the standard sample size formula [4],



where *p *= (*p*_*C *_+ *p*_*I*_)/2, 1.644485 is the z-statistics corresponding to the 95th percentile of the normal distribution (for a one-sided type I error of .05) and 1.28155 is the z-statistics corresponding to the 90th percentile (for a power of .90). Based on (4), the sample size for a trial using average-risk subjects from the general population study is 2529 per group and the sample size for a trial of high-risk subjects is 1244 per group. Let *C*_*R *_denote the cost of recruitment per subject and *C*_*I *_denote the cost of intervention and follow-up per subject *averaged over the two randomization groups*. Suppose high risk subjects comprise a fraction *f *of the general population. The total cost of the trial for average-risk subjects from the general populations is

*C*_general _= 2(*C*_*R *_2529 + *C*_*I *_2529),    (5)

and the total cost of the trial for high-risk subjects is

*C*_high-risk _= 2(*C*_*R *_1244/*f *+ *C*_*I *_1244).    (6)

where the factor of 2 is for the two randomization groups. The condition for the trial of high-risk subjects to cost more than the trial of average-risk subjects (namely *C*_high-risk _>*C*_general_) is



when 1244/*f *- 2529 > 0. If *f *= .20, the trial of high-risk subjects will cost more than the trial of average-risk subjects if *C*_*R*_/*C*_*I *_> .34. If *f *= .10, the trial of high-risk subjects will cost more than the trial of average-risk subjects if *C*_*R*_/*C*_*I *_> .13.

In many cancer prevention trials the above values of *C*_*R*_/*C*_*I *_are likely. For example, diagnostic testing to identify high-risk smokers can include expensive airway pulmonary function tests or bronchoscopy. In the future, more trials will likely involve expensive genetic testing of subjects [5] with costs ranging from $350 to almost $3,000 per test according to recent information from Myriad Genetic Laboratories. As part of a sensitivity analysis related to genetic testing of subjects prior to enrollment in a trial, Baker and Freedman [5] considered values of .1, .5, and 1 for ratios similar to *C*_*R*_/*C*_*I*_.

Even without diagnostic testing, the costs of obtaining high-risk subjects can be substantial. If *f *= .10, the initial recruitment will require ten times the number of people as for a trial of average-risk subjects from the general population. This increased recruitment would likely require higher advertising costs and increased overhead costs from the inclusion of additional institutions.

One additional consideration is how noncompliance and contamination affect the intent-to-treat analysis. If noncompliance and contamination can be anticipated, the investigator can correspondingly adjust the sample size and costs. Mathematically the effect of noncompliance and contamination is to change the values of *p*_*C *_and *p*_*I *_in (4), which would then affect (5) and (6). In some settings, investigators may anticipate that high-risk subjects are more likely to comply with the intervention than average-risk subjects. To compensate for the anticipated increased compliance, study designers could reduce the sample size which would lower costs. However, in other situations, investigators may anticipate that subjects found to be at high-risk on a diagnostic test would likely seek the best therapy outside of the trial rather than chance randomization to standard or experimental therapy. To compensate for the anticipated dilution in treatment effect, investigators would need to increase the sample size which would increase the costs.

For the above reasons even if the probabilities under the alternative hypothesis are correctly specified, some trials of high-risk subjects may be more expensive than larger trials of average-risk subjects with the same power and type I error.

### Possibly misleading ratio of benefits to harms

When there is strong evidence prior to the trial of a high probability of harmful side effects due to the intervention, one would want to restrict the intervention to high-risk subjects. Otherwise, some investigators may be tempted to estimate the ratio of benefit to harms in the trial of high-risk subjects and extrapolate the ratio to average risk subjects. Unfortunately, even if the assumption of constant relative risk over risk categories were true, extrapolating the benefit-harm ratio from a high risk group to the general population could be misleading.

Suppose that in a randomized trial involving average-risk subjects from the general population the probability of cancer is .02 in the control arm and .01 in the study arm. Also suppose that relative risk is same in the general population as in the high-risk group, so that in a randomized trial involving a high-risk group, the probability of cancer is .04 in the control arm and .02 in the study arm. Furthermore, suppose that the probability of harmful side effects is the same for high-risk subjects as for average-risk subjects in the general population, namely .015 in the control arm and .025 in the study arm. Based on these results, for every 1000 high-risk persons who receive the intervention, (.04 - .02) 1000 = 20 will benefit from the intervention and (.025 - .015) 1000 = 10 will be harmed by side effects, yielding a benefit-harm ratio of 20:10 = 2:1. Similarly for every 1000 average-risk person who receive the intervention, (.02 - .01) 1000 = 10 will benefit from the intervention and (.025 - .015) 1000 = 10 will be harmed by side effects yielding a benefit-harm ratio of 10:10 = 1:1. In this example it would be incorrect to extrapolate the high benefit-harm ratio estimated from the high-risk group to the general population for whom the benefit-harm ratio is much lower. For many cancer prevention interventions, the ratio of life-threatening disease avoided to life threatening harms would be favorable in the high-risk group but not favorable when extrapolated to the general population.

## Conclusion

There is no "free lunch" when using high-risk subjects in prevention trials design to make inference about the general population. Using high risk subjects instead of average-risk subjects from the general population may lower statistical power, increase costs, and yield a misleading ratio of benefit to harms than actually the case.

Given the substantial costs of definitive randomized trials in cancer prevention, and the importance of accurately assessing the balance of benefit and harm when treating healthy and asymptomatic people, it is therefore important to conduct trials in the actual target population rather than try to conduct them in high-risk populations with the plan to extrapolate results to the general population.

## Competing Interests

The authors declare that they have no competing interests.

## Authors' contributions

SGB wrote the initial draft, and BSK and DC made valuable improvements. All authors read and approved the final manuscript.

## Pre-publication history

The pre-publication history for this paper can be accessed here:



## Supplementary Material

Additional File 1 Appendix A, worked-out calculations of power. Click here for file

Additional File 2 Appendix B, likelihood formulations Click here for file
