# Supplementary material for: The fallacy of enrolling only high-risk subjects in cancer prevention trials: Is there a "free lunch"?
Source: BMC Med Res Methodol. 2004 Oct 4;4:24. doi: 10.1186/1471-2288-4-24 (PMC524373; doi:10.1186/1471-2288-4-24)
Supplement: Additional File 1 — Appendix A, worked-out calculations of power. [file 1471-2288-4-24-S1.pdf]

Appendix A.

Worked-out calculations of power. The calculations are based on more significant digits than are displayed.

Power with $p_C = .02$ and $p_I = .01$

Risk Difference

$\Delta_1 = .02 - .01 = .01,$

$p = (.02 + .01)/2 = .015$

$se_{1Null} = \sqrt{2\,(.015)\ (1 - .015)} = .1719$

$se_{1Alt} = \sqrt{.02\,(1\text{-}.02) + .01\,(1\text{-}.01) =}\ .1718$

$z = (.01 / \sqrt{2000} - 1.96 \times .1719)/\,.1718 = .642$

power = NormalCDF(.642) = .74

Relative risk

$\Delta_2 = \text{Log}(.02/.01) = .69,$

$p = (.02 + .01)/2 = .015$

$se_{2Null} = \sqrt{2\ (1 - .015)\ /.015} = 11.46$

$se_{2Alt} = \sqrt{(1\text{-}.02)/.02 + (1\text{-}.01)/.01} = 12.17$

$z = (.69\sqrt{2000} - 1.96\ 11.46)/12.17 = .70$

power= NormalCDF(.70)=.76

Power with $p_C = .04$ and $p_I = .02$

Risk Difference

$\Delta_1 = .04 - .02 = .02,$

$p = (.04 + .02)/2 = .03$

$se_{1Null} = \sqrt{2\,(.03)\ (1 - .03)} = .24$

$se_{1Alt} = \sqrt{.04\,(1\text{-}.04) + .02\,(1\text{-}.02) =}\ .24$

$z = (.02 / \sqrt{2000} - 1.96 \times .26) / .26 = 1.75$

power = NormalCDF(1.75) = .96

Relative risk

$\Delta_2 = \text{Log}(.04/.02) = .69,$

$p = (.04 + .02)/2 = .03$

$se_{2Null} = \sqrt{2 (1 - .03) / .03} = 8.04$

$se_{2Alt} = \sqrt{(1\text{-}.04)/.04 + (1\text{-}.02)/.02} = 8.54$

$z = (.69 \sqrt{2000} - 1.96\ 8.04)/8.54 = 1.78$

power= NormalCDF(1.78)=.96

Power with $p_C = .04$ and $p_I = .03$

Risk Difference

$\Delta_1 = .04 - .03 = .01,$

$p = (.04 + .03)/2 = .035$

$se_{1Null} = \sqrt{2 (.035) (1 - .035)} = .26$

$se_{1Alt} = \sqrt{.04 (1\text{-}.04) + .03 (1\text{-}.03)} = .26$

$z = (.01 / \sqrt{2000} - 1.96 \times .26) / .26 = \text{-}.2394$

power = NormalCDF(-.2394) = .41

Relative risk

$\Delta_2 = \text{Log}(.04/.03) = .288,$

$p = (.04 + .03)/2 = .035$

$se_{2Null} = \sqrt{2 (1 - .035) / .035} = 7.42$

$se_{2Alt} = \sqrt{(1\text{-}.04)/.04 + (1\text{-}.03)/.03} = 7.51$

$z = (.288 \sqrt{2000} - 1.96\ 7.42)/ 7.51 = \text{-}.225$

power= NormalCDF(-.225)=.42
