# Supplementary material for: The fallacy of enrolling only high-risk subjects in cancer prevention trials: Is there a "free lunch"?
Source: BMC Med Res Methodol. 2004 Oct 4;4:24. doi: 10.1186/1471-2288-4-24 (PMC524373; doi:10.1186/1471-2288-4-24)
Supplement: Additional File 2 — Appendix B, likelihood formulations [file 1471-2288-4-24-S2.pdf]

Appendix B

Let n_{ij0} denote the number in risk group i ($i = 1, 2, ..., k$) and arm j ($j = 0$ = placebo, $j = 1$ = tamoxifen) without invasive breast cancer. Let n_{ij1} denote the total number in risk group i and arm j with invasive breast cancer. The likelihood kernal for the general formulation is

$$L = \prod_i (1 - pr(\text{invasive breast cancer} \mid \text{placebo, group } i))^{n_{i00}}$$
$$\times\ pr(\text{ invasive breast cancer} \mid \text{placebo, group } i)^{n_{i01}}$$
$$\times (1 - pr(\text{invasive breast cancer} \mid \text{tamoxifen, group } i))^{n_{i10}}$$
$$\times\ pr(\text{ invasive breast cancer} \mid \text{tamoxifen, group } i)^{n_{i11}}.$$

Recall that $\pi_i = pr(\text{invasive breast cancer} \mid \text{placebo, group } i)$. Let δ denote the absolute risk difference which is constant over risk groups. The kernel of the log-likelihood for the *Constant RD* model is

$$L_{ConstantRD}(\pi_i, \delta) = \sum_{i=1}^{k} n_{i00}\, Log\,(1 - \pi_i) + \sum_{i=1}^{k} n_{i01}\, Log\,(\pi_i)$$
$$+ \sum_{i=1}^{k} n_{i10}\, Log\,(1 - \pi_i + \delta) + \sum_{i=1}^{k} n_{i11}\, Log\,(\pi_i - \delta)\,.$$

Let β denote the relative risk which is constant over risk groups. The kernel of the log-likelihood for the *Constant RR* model is

$$L_{ConstantRR}\,(\pi_i, \beta) = \sum_{i=1}^{k} n_{i00}\, Log\,(1 - \pi_i) + \sum_{i=1}^{k} n_{i01}\, Log\,(\pi_i)$$
$$+ \sum_{i=1}^{k} n_{i10}\, Log\,(1 - \pi_i/\beta) + \sum_{i=1}^{k} n_{i11}\, Log\,(\pi_i/\beta).$$

The above log-likelihoods were maximized using a Newton-Raphson algorithm with starting values of $\pi_i = n_{i11}/n_{i1+}$, $\delta = \Sigma_i(n_{i01}/n_{i0+} - n_{i11}/n_{i1+})/k$, and $\beta = \Sigma_i((n_{i01}/n_{i0+})$ / $(n_{i11}/n_{i1+}))/k$, where "+" indicates summation over the indicated subscript. Confidence intervals are based on the asymptotic variance computed via the observed information matrix.

For the full model the estimates are $\widehat{\delta}_i = n_{i01}/n_{i0+} - n_{i11}/n_{i1+}$ and $\widehat{\beta}_i = (n_{i01}/n_{i0+})$ / (n_{i11}/n_{i1+}). Confidence intervals are based on the asymptotic variance for binomial distritubutions. The maximized log-likelihood for both *Varying RD* and *Varying RR* models is

$$
\begin{aligned}
L_{VaryingRD}(\widehat{\pi}_i, \widehat{\delta}_i) = L_{VaryingRR}(\widehat{\pi}_i, \widehat{\beta}_i) = \\
\sum_{i=1}^{k} n_{i00} Log(n_{i00}/n_{i0+}) + \sum_{i=1}^{k} n_{i01} Log(n_{i01}/n_{i0+}) \\
+ \sum_{i=1}^{k} n_{i10}\, Log\,(n_{i10}/n_{i1+}) + \sum_{i=1}^{k} n_{i11}\, Log(n_{i11}/n_{i1+}).
\end{aligned}
$$

Based on an asymptotic chi-squared distribution, p-values for comparing models are computed for $2\,(L_{VaryingRD}(\widehat{\pi}_i, \widehat{\delta}_i) - L_{ConstantRD}\,(\widehat{\pi}_i, \widehat{\delta}))$ and $2\,(L_{VaryingRR}(\widehat{\pi}_i, \widehat{\beta}_i) - L_{ConstantRD}\,(\widehat{\pi}_i, \widehat{\beta}))$.
